# Ancient Venom Systems: A Review on Cnidaria Toxins

**DOI:** 10.3390/toxins7062251

**Published:** 2015-06-18

**Authors:** Mahdokht Jouiaei, Angel A. Yanagihara, Bruno Madio, Timo J. Nevalainen, Paul F. Alewood, Bryan G. Fry

**Affiliations:** 1 Venom Evolution Lab, School of Biological Sciences, the University of Queensland, St. Lucia 4072, QLD, Australia; E-Mail: m.jouiaei@uq.edu.au; 2 Institute for Molecular Bioscience, the University of Queensland, St. Lucia 4072, QLD, Australia; E-Mails: b.madio@uq.edu.au (B.M.); p.alewood@imb.uq.edu.au (P.F.A.); 3 Pacific Cnidaria Research Lab, Department of Tropical Medicine, University of Hawaii, Honolulu, HI 96822, USA; E-Mail: ayanagih@hawaii.edu; 4 Department of Pathology, University of Turku, Turku FIN-20520, Finland; E-Mail: timneva@utu.fi

**Keywords:** cnidarians, venom, enzymes, pore forming toxins, neurotoxins, vasodilatory biogenic amines, human envenomation

## Abstract

Cnidarians are the oldest extant lineage of venomous animals. Despite their simple anatomy, they are capable of subduing or repelling prey and predator species that are far more complex and recently evolved. Utilizing specialized penetrating nematocysts, cnidarians inject the nematocyst content or “venom” that initiates toxic and immunological reactions in the envenomated organism. These venoms contain enzymes, potent pore forming toxins, and neurotoxins. Enzymes include lipolytic and proteolytic proteins that catabolize prey tissues. Cnidarian pore forming toxins self-assemble to form robust membrane pores that can cause cell death via osmotic lysis. Neurotoxins exhibit rapid ion channel specific activities. In addition, certain cnidarian venoms contain or induce the release of host vasodilatory biogenic amines such as serotonin, histamine, bunodosine and caissarone accelerating the pathogenic effects of other venom enzymes and porins. The cnidarian attacking/defending mechanism is fast and efficient, and massive envenomation of humans may result in death, in some cases within a few minutes to an hour after sting. The complexity of venom components represents a unique therapeutic challenge and probably reflects the ancient evolutionary history of the cnidarian venom system. Thus, they are invaluable as a therapeutic target for sting treatment or as lead compounds for drug design.

## 1. Introduction

The phylum Cnidaria (corals, sea pens, sea anemones, jellyfish and hydroids) includes about 10,000 species living in aquatic habitats worldwide [1]. They range in size from the tiny *Hydra* spp, at less than 1 cm in length, to the massive lion’s mane jellyfish, *Cyanea capillata* with the bell diameter exceeding 2 m [2]. Envenomation hazard to humans also varies widely from non-hazardous to the infamous *Chironex fleckeri* (Australian box jellyfish), one of the most venomous animal dangerous to humans, as a meter of tentacle contact can provoke immediate cardiovascular collapse and death even within minutes after a sting [3]. The majority of cnidarians live in salt water habitats at different water depths. However, approximately 40 species, mostly hydrozoans [4] live in freshwater. Cnidarians are characteristically radially symmetrical [5], although they can also exhibit directional asymmetry or bilateral symmetry. For example, morphological studies on Siphonophores (class Hydrozoa) suggest that directional asymmetry has been gained and/or lost on multiple occasions [6], whilst most anthozoan polyps exhibit bilateral symmetry possessing two orthogonal body axes [5].

Despite the variety in size, toxicity, habitat and morphology several cellular characters are common to the members of Cnidaria, such as two unicellular layers (ectoderm and endoderm) separated by an extra-cellular matrix (mesoglea), neuromuscular systems and multiple sensory systems [7,8]. Molecular evidence and fossil data place the origin of cnidarins prior to the Ediacaran period ~750 million years ago, and major taxa diversification from the remaining metazoans prior to the Cambrian ~550 million years ago [9,10,11].

Since Cnidaria is an ancient clade of animals and the complexity and diversification of their venoms serve a unique therapeutic challenge (e.g., box jellyfishes (Cubozoa) venoms), transcriptomics and proteomics data for the identification and characterizing of their venom components is rapidly accumulating in recent times [12,13,14].

## 2. Cnidarian Phylogeny

Based upon mitochondrial DNA (mtDNA) data [15] and life cycles [8,16], cnidarians are divided into two extant subphyla: Anthozoa and Medusozoa. Anthozoans possess circular mtDNA, similar to that of other metazoans while medusozoans have atypical linear mtDNA. The members of medusozoan classes Hydrozoa, Schyphozoa, Cubozoa and Staurozoa display a triphasic life cycle in transition of generations: a free-swimming planula larva, a sessile polyp stage and sexual pelagic medusa stage. In anthozoans the medusa stage is lost and sessile adults represent the sexually propagating stage. The life cycle will be discussed in more details further in this review.

## 3. Cnidarian Life Cycle

There is significant morphological diversity in the cnidarian life cycle, as a single species may display a variety of forms whether it is sessile, polyp, tiny free-swimming planula larva or a pelagic medusoid. The life cycle of both medusozoans and anthozoans comprises sexual reproduction and an asexually budding phase. In medusozoans, the adult medusa is either male or female, and the fertilized egg (zygote) is retained inside the female’s gastric cavity [17,18]. However, in anthozoans, the polyp colonies may be single sex [19] or both male and female [20]. In general, the asexual life cycle of medusozoans includes a fertilized egg, which forms tiny pelagic planula larva that settles down to the sea floor and form a sessile polyp. These polyps further develop a hydroid polyp colony, which liberates medusae by budding from the trunk [18]. Amongst the medusozoans, hydrozoans have the greatest variation in life cycle. For example, species in the Campanulariidae family lack the medusa stage [21] and the members of the order Trachymedusae never form polyps [16]. The asexual life cycle of anthozoans is straightforward including four main stages: the fertilized egg, planula larvae, polyp and sessile sea anemone [16,22].

## 4. Cnidarian Venom Delivery System

**Figure 1:**
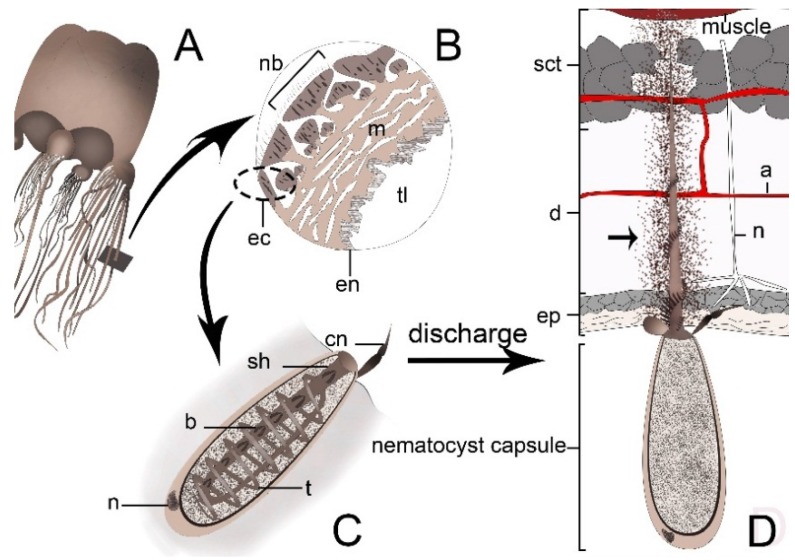
Overview of the Cnidarian venom delivery system. (**A**) Schematic picture of a jellyfish; (**B**) Transverse section of a tentacle showing two epithelial cell layers, ectoderm (ec) and endoderm (en), divided by the mesoglea (m), various types of cnidocytes placed in nematocyst batteries (nb) and in the cells lining the tentacular lumen (tl); (**C**) Undischarged nematocyst. The nematocyst capsule contains a cocktail of toxins, shaft (sh), coiled-hollow tubule (t), barbs (b), and nucleus (n). The cnidocil (cn) acts as a mechanoreceptor that upon activation stimulates the discharge mechanism; (**D**) Discharged nematocyst. Epidermis (ep), dermis (d), subcutaneous tissue (sct), artery (a), nerve (n). Toxin mixture is injected into the prey’s skin and subcutaneous tissues (arrow). The figure was drawn according to the data in references [14,29,34,35].

Cnidae are the defining subcellular specialisation of the phylum Cnidaria. They are specialized cellular structures capable of explosive discharge upon activation of cnidocytes (Figure 1). Cnidae contain elaborate structural elements and complex mixtures of bioactive compounds or “venom” for entrapping, subduing and digesting prey as well as deterring and repelling predators and competitors [23]. Cnidae are distributed in various parts of the cnidarian body and are classified into three major types: penetrant nematocysts, the volvent spirocyst and the glutinant ptychocysts.

Cnidae comprise an eversible hollow tubule that is coiled and sometime spine laden. These phylum-specific organelles are synthesized in specialised precursor cells called cnidoblasts. Following their secretion from the Golgi apparatus, the cnidae undergo further structural modifications in the extracellular matrix before migrating to the tentacle surface [24,25,26].

Penetrant nematocysts inject venom into the target organism and are the most studied class of cnidae. Penetrant nematocysts are found in all cnidarians and are morphologically and functionally the most diverse group of cnidae [27]. They are the primary weapon for capturing prey, repelling predators, and intra- and interspecies spatial competition [28]. Although nematocysts are mostly located on the tentacles, they also exist on the outer surface of the bell in certain species of the Alatinidae, and Carybdeidae families [29], oral arms of *Catostylus mosaicus* (Schyphozoa) [30] and the stomach (gastric cirri) of some cubozoans [29] probably helping to paralyse and digest the prey. Members of family Actiniidae also have nematocysts in the ring around the base of their tentacles, called acrorhagi, which they use for intra- and interspecific competition [31] and at filaments called acontia that are used for defence or paralysis of prey.

Spirocysts are present in most anthozoans and may differ morphologically among different taxonomic groups [32]. The spirocyst capsule wall is thin and the everted tubule is helically folded. The tubule lacks spines, but contains adhering, hydroscopic substances that mechanically immobilize the prey [33]. Ptychocysts have been reported in tube-dwelling members of Actinaria and Ceriantharia. They create the protective tube in which species from these families live [33].

The mechanism of cnidae discharge in response to external stimuli (mechanical and chemical stimuli) is still not completely understood. The general osmotic/tension hypothesis proposes that the osmotic pressure of the intracapsular fluid temporary increases as a result of cnidae exposure to the external solution and subsequent exocytosis of cations from the capsule. The osmotic pressure differences across the capsule wall extant to the point that the preexisting intracapsular pressure exceeds a critical threshold value and triggers the cnidae discharge [36,37,38,39]. The capsule content (venom) is located on the inner surface of the inverted tubule which, during discharge (tubule eversion-extension), everts such that the outside is now exposed and injected into the prey. The inside of the tubule remains contiguous with the inside of the capsule and the cnidae content or venom is then expelled down the axis of the hollow tubule [40].

The morphological properties of cnidae are taxonomically informative and may aid in distinguishing more distantly related species [33]. For instance, the presence of spirocyst is characteristic of some anthozoans [22]. In addition, the relative abundance of different types of nematocyst, along with variation in the body size in several populations may correlate to environmental factors, such as specific ecosystems, prey size and selectivity in prey capture. For example, *Chironex fleckeri* has a relatively large body and a higher ratio of mastigophores (the longest of the penetrant cnidae) correlating to the large organisms which they prey upon [41]. In addition to cnidae, venom of Cnidarians was shown to be also synthesized in other cell types such as ectodermal and endodermal gland cells and other, still unknown, cell types [42,43].

**Table 1:** Cnidarian venom composition.

Toxin type	Found in class	Function	MW (kDa)	Reference
**Enzymes**				
Phospholipase A_2_	Anthozoa, Cubozoa, Schyphozoa, Hydrozoa	Cytolytic, hemolytic, prey digestion	13–45	[44,45,46]
Metalloproteases	Schyphozoa, Cubozoa, Anthozoa	Cytotoxic, cytolytic, local tissue damage	17–130	[12,14,47,48]
**Pore forming toxins (cytolysins)**				
Actinoporins and actinoporin-like proteins	Anthozoa, Hydrozoa	Cytolytic, hemolytic, cardiovascular and respiratory arrest	20	[49,50,51,52,53,54]
Jellyfish Toxins	Cubozoa	Hemolytic, cardiotoxic, cytolytic, myotoxic, cutaneous inflammation	42–46	[13,55,56,57]
Hydralysins-related toxins	Hydrozoa, Anthozoa	Cytolytic, prey digestion	27–31	[43,58,59,60]
Membrane Attack Complex-Perforin	Anthozoa	Cytolytic, hemolytic	60	[61,62]
**Neurotoxins**				
NaTxs (type I-III)	Anthozoa	Neurotoxic, cardiotoxic, insecticide	3–8	[63,64,65,66,67]
KTxs (type I,III and IV,V KTxs)	Anthozoa	Neurotoxic, hypotensive, cardiotoxic, analgesic, antimicrobial, immunosuppressive, anti obesity	3–4	[68,69,70,71,72,73,74,75]
Kunitz peptides (type II KTxs)	Anthozoa	Paralytic, serine protease inhibitor	6	[76,77,78,79]
Small Cysteine-Rich Proteins (SCRiPs) and SCRiPs homologues	Anthozoa	Paralytic	4.3–5.8	[80]
ASIC Inhibitors	Anthozoa	Analgesic	3	[81,82]
TRPV1 Inhibitors	Anthozoa	Analgesic	3	[83,84]
**Non-protein bioactive components**				
Serotonin	Hydrozoa, Anthozoa	Vasodilation, sharp pain	-	[85]
Histamine	Anthozoa	Vasodilation, sharp pain	-	[86,87]
Bunodosine	Anthozoa	Analgesic	-	[88]
Caissarone	Anthozoa	Adenosine receptor antagonist	-	[89]

## 5. Venom Composition

Since the early 20th century, numerous analytical experiments and clinical observations have established the toxicological diversity of cnidarian venoms (Table 1). The diversity of venom components range from non-proteinaceous compounds (e.g. purines, biogenic amines) to high molecular weight proteins evolved over the course of hundred million years. Interestingly, some toxin family types identified previously in other venomous animals comprise the venom arsenal of cnidarians. The most striking example is Kunitz peptides are expressed in sea anemones, cone snails, insects, scorpions, spiders, reptiles, ticks and vampire bat [90,91]. Another example of convergent expression of toxins is potassium channel blocker Kv1 which not only evolved convergently in scorpions and sea anemones, but they even use the same residues for blocking the channel pores [92].

### 5.1. Enzymes

#### 5.1.1. Phospholipase A_2_

Phospholipase A_2_ (PLA_2_) activity has been detected in the homogenized tentacles and acontia of cnidarians including subphylums Anthozoa, Schyphozoa, Hydrozoa and Cubozoa [44,46,93]. This enzyme hydrolyzes the *sn*-2 acyl bond of glycerophospholipids to produce fatty acids including arachidonic acid and lysophospholipid [22,44]. It is commonly found in mammalian tissues where it plays important roles in vital body functions including dietary lipid catabolism, inflammation, signal transduction, and phospholipid remodelling [94]. However, the ubiquitous presence of PLA_2_ across venomous animal lineages such as reptiles (snakes and anguimorph lizard), centipedes, insects (their bristles, proboscises, and stingers), arachnids (scorpions, spiders, and ticks), cnidarians, and cephalopods [44,90,95,96,97,98] indicates the convergent recruitment of the body proteins into the toxic arsenal of animals. Toxic functions of PLA_2_ in cnidarian venoms have been proposed to include defence and immobilization and digestion of prey [44,93]. Also, hemolytic activity was demonstrated in a PLA_2_ fraction recovered from acontial nematocysts of the sea anemone *Aiptasia pallida* [45]. Although PLA_2_ enzymes are plentiful amongst cnidarians [44,98], their molecular structure-function relationships remain to be elucidated.

#### 5.1.2. Metalloproteases

Metalloproteases are important venom components of terrestrial animals such as centipedes, snakes, and ticks [90,99]. They induce haemorrhage and necrosis by degrading the extracellular matrix and preventing blood clot formation [90,100]. These functions are commonly associated with several of the recurrent symptoms of sting such as skin damage, oedema, blister formation, myonecrosis and inflammation [100]. Metalloproteases were detected in the venom of jellyfish *Stomolophus meleagris* [12] and *Chironex fleckeri* [14]. A recent study focused on the enzymatic and cytotoxic functions of jellyfish metalloproteases and identified diverse proteolytic effects including gelatinolytic, caseinolytic, and fibrinolytic activities [47]. Zinc-dependent metalloproteases of the astacin family were detected in the soluble nematocyst content of the sea anemone *Nematostella vectensis* and were shown to be expressed in both gland cells and stinging cells. They originate from recruitment of the Bone Morphogenic Protein 1 (BMP1, also known as Tolloid) protein that plays a conserved role in animal development [48].

### 5.2. Pore Forming Toxins

Pore forming toxins (PFTs) appear to be present in all cnidarian venoms. The mechanism of action of these toxins is penetration through the target cell membrane resulting in diffusion of small molecules and solutes leading to osmotic imbalance and cell lysis. PFTs exhibit dual structure (i) a stable water-soluble structure that is monomeric and binds to the receptors on the target cell; (ii) membrane-bound structure consisting of oligomeric moleculs that form integral membrane pores [101]. Cnidarian PFTs are classified in two groups based on the type of secondary structure they use to penetrate membrane upon pore-forming activity: α-PFTs where they are a rich content of helices and form α-helical barrel structures, and β-PFTs that are rich in β-sheets and form β-barrel pores.

#### 5.2.1. Actinoporins

Actinporins are α-PFTs and are present in Anthozoa and Hydrozoa. They are basic proteins approximately 20 kDa in molecular size lacking in intramolecular disulfide bonds. Actinoporins mediate various types of toxicity and bioactivity, such as cardiovascular and respiratory arrest in rats [50], lysis of chicken, goat, human and sheep erythrocytes [51,53,54] and cytotoxic effects [52], all caused by a pore-forming mechanism. Several studies have reported that actinoporins interact exclusively with sphingomyelin containing membranes [102,103,104], although sticholysin II from Stichodactyla helianthus binds to phosphatidylcholine membrane [105]. The mechanism of membrane penetration requires several steps: (i) initial binding to the target membrane which is accomplished by an exposed aromatic-rich loop; (ii) lateral orientation and consequent oligomerization by a phosphocolin binding site; and (iii) insertion of the *N*-terminal amphiphilic α-helix segment to the lipid membrane [102,103,106]. High-resolution nuclear magnetic resonance and Fourier transform infrared spectroscopy experiments [107], intrinsic fluorescence measurements [108], X-ray crystallography and electron microscopic analyses of two dimensional crystals [109] have suggested that, during the membrane penetration, the *N*-terminal amphiphilic α-helix segment of actinoporins detach from the main body and insert into the target membrane to produce pores with a diameter of 1–3 nm (~11–30 Å) [54,110].

#### 5.2.2. Jellyfish Toxins (JFTs)

Cubozoan-related porins are the most potent and rapid-acting toxins secreted by jellyfish species. This toxin family was originally reported in the cubozoan *Carybdea alata* as CAH1 [111], also reported as CaTX-A/B [112], and subsequently identified in all cubozoans examined including CrTX-A/B from *Carybdea rastoni* [55], CqTX-A from *Chiropsalmus quadrigatus* [56], CfTX-1/2 and CfTX-A/B/Bt from *Chironex fleckeri* [13,113]. However, homologues of cubozoan porins were reported in Scyphozoa (*Aurelia aurita*), Hydrozoa (*Hydra magnipapillata*) [13], Anthozoa (*Aiptasia pallida*) and various hydroids (*Hydractinia symbiolongicarpus* and *Hydra vulgaris*), suggesting a common evolutionary origin of these toxins [80]. Characteristically, they are basic proteins with a molecular weight of 40 to 46 kDa and contain both α and β domains. The hypothetical mechanisms underlying pore formation involves oligomerization of several amphiphilic and hydrophobic α-helices in the *N*-terminal region of the toxin resulting in distortion of the plasma membrane and cell death [13,112,113]. The similarity in the three-dimensional structure of the *N*-terminal domain of CfTX toxins to that of the α-pore-forming domain (domain I) of the insecticidal δ-endotoxins from *Bacillus thuringiensis* suggests how toxins of this family insert into membranes. This mechanism of membrane insertion seem to be sufficient to explain the well-defined 12 nm (inner) and 25 nm (outer) diameter pores created in human erythrocytes by CfTX toxins [3].

Interestingly, the members of this toxin family exhibit varying target specify towards various vertebrate tissue. A recent study on CfTX toxins showed that CfTX-1/2 caused cardiovascular collapse within 1 min in anesthetized rats exposed *in vivo* to the venom, whereas CfTX-A/B more potent in eliciting *in vitro* hemolytic activity [13]. Although these data suggest that the jellyfish toxin family has undergone functional diversification, more structural/functional data are needed to understand the molecular evolutionary histories that formed this diversification.

#### 5.2.3. Hydralysins-Related Toxins

In addition to porins found in cnidarians, recent studies have described a novel β-PFT family secreted from the digestive endodermal cells of green hydra (*Chlorohydra* *viridissima*) [43,58]. These non-nematocyst, body-derived toxins secreted during feeding are suggested to play a role in lysing prey tissues [59]. These toxins are not active on membrane phospholipids or carbohydrates, but rather bind to specific membrane receptors and form pores [58]. A recent study characterized aerolysin, a hydralysin homologue, secreted from pharyngeal ectodermal cell of *N. vectensis* and suggested a role in prey disintegration [60].

#### 5.2.4. Membrane Attack Complex-Perforin

This group of β-PFT toxins have been detected in the venoms extracted from the sea anemones *Phyllodiscus semoni* and *Actinaria villosa*. The toxins may be used in prey capture/predator defence [61,62]. Membrane attack complex (MAC) proteins have been identified in the complement system produced by T-cells and NK cells. The proteins create a transmembrane pore into the target cell and initiate various apoptotic cell death pathways [114].

### 5.3. Neurotoxins

Cnidarian neurotoxins (voltage-gated ion channel toxins) are a group of low molecular weight peptides and are among the best characterized toxins in terms of the mechanism of action. They are produced by sea anemones and have a fundamental role in the venom to help these sessile animals to immobilize the prey rapidly and to defend against predators. They prolong the action potential of the excitable and non-excitable membranes in sensory neurons and cardiac and skeletal muscle cells [64,115] via modifying the sodium channel gating [64,116,117] or blocking the potassium channel gating during the repolarisation stage [115]. This causes the cell to become hyperactive and to release massive amounts of neurotransmitter at synapses and neuromuscular junctions that can cause initial spastic stage followed by descending flaccid paralysis. Sea anemone voltage-gated ion channel toxins have been studied extensively because they are valuable bioresources to study the structure and function of sodium and potassium channels and also to be used for development of drugs [73,74] and bioinsecticides [66,67].

Several cnidarian neurotoxin polypeptides are exclusively block Vanilloid Receptor 1 (TRPV1) and acid-sensing ion channel 3 (ASIC3), which take part in initiation and transduction of pain and hyper-algesia. These peptides are thus promising tools for the development of novel pain reducers.

#### 5.3.1. Voltage-Gated Sodium (Nav) Channel Toxins

Voltage-gated sodium (Nav) channel toxins (NaTxs) are transmembrane complexes composed of several subunits. The highly conserved α-subunit consists of four homologous domains (D1–D4) each containing six hydrophobic transmembrane regions (S1–S6) [117]. Anthozoan NaTxs and several other groups of toxins from scorpion and spiders bind to site 3 (loop S3–S4 in D4) of the Nav channels [64,118] resulting in considerable neurotransmitter release in synapses. One possible explanation underlying its toxicity is the electrostatic interaction between a cluster of basic amino acids on the toxin with acidic amino acids at site 3 [119] locking S4 segment in its inward position, thus inhibiting the conformational changes of the channel necessary for fast inactivation [117]. These toxins are divided into three groups: (i) type I NaTx; and (ii) type II NaTxs exhibit extensive sequence similarity and share similar function. In addition to type I and II NaTxs there is an orphan NaTx clade, that exhibits only a partial homology to both type I and type II NaTxs and shows similar mechanism of action to those [120,121]. Lastly, there are short (~30 amino acids long) peptides in sea anemone venom that exhibit a similar activity to the rest of NaTxs families despite lacking any shared sequence motifs with the rest of them [118]. These peptides form the type III family and exhibit very high selectivity towards arthropod sodium channels [122].

#### 5.3.2. Voltage-gated Potasium (Kv) Channel Toxins and Kunitz peptides

Sea anemone voltage-gated potasoim (Kv) channel toxins (KTxs) are categorized in five groups based on their sequence similarity and binding affinity towards different Kv channel families [72]. Type I KTxs have a molecular weight of 4 kDa with three disulfide bridges. They inhibit the potassium current through channel Kv1. and Kv3. subfamilies and intermediate conductance calcium-activated potasoim channels [22]. Type II KTxs are Kunitz peptides with a molecular weight of 6 kDa that are cross-linked with three disulfide bridges and are remarkable in their dual function of activity. Usually they act against trypsin and chymotrypsin proteinases in order to inhibit the rapid degradation of the venom protease by endogenous enzymes of the animals themselves or of the prey [31,78,79]. Interestingly, several sea anemone Kunitz peptides possess both Kv blocking activity similar to dendrotoxin and protease inhibiting activity [76,123]. For example, kalicludin 1-3 from *A. sulcata* binds competitively to Kv1.2 channels to paralyse the prey rapidly. Type III KTxs are 3–4 kDa peptides with three disulfide bridges that have evolved from NaTxs under the regime of positive selection [80]. They block a variety of distinct potassium ion channels such as Kv3.4 channel belonging to the rapidly inactivating Kv channel [68] and hERG (the human Ether-à-go-go-Related Gene) [69,75]. Interestingly, the influence of positive selection does not result in the complete loss of sodium ion channel blocking activity, where several type III KTxs target both hERG and acid-sensing ion channel 3 (ASIC3, H^+^-gated Nav channels) [75]. Type IV are structurally new peptides from the sea anemone *Stichodactyla haddoni* displaying crab paralyzis activity and are cross-linked with two disulfide bridges [124]. Type V has been found in the sea anemone *Bunodosoma caissarum* and is cross-linked by four disulfide bridges. This novel peptide is active on Drosophila Shaker IR channels [72].

#### 5.3.3. Small Cysteine-Rich Peptides (SCRiPs)

This newly discovered group of neurotoxins have been reported in the ectoderm of reef-building corals *Acropora millepora* and SCRiP homologs have been retrieved in sea anemones, *Anemonia viridis* and *Metridium senile* [80]. The injection of recombinantly expressed *A. millepora* SCRiPs in zebrafish (*Danio rerio*) larvae resulted in severe paralysis, suggesting the first peptide neurotoxin family described from scleractinian corals [80].

#### 5.3.4. ASIC Inhibitors

ASICs are sodium-selective acid-sensing ion channels (ASIC) expressed in peripheral neuronal system. They have been associated with acidic pain during pathological conditions such as inflammation and ischemia. Recently, a novel peptide π-AnmTX Ugr 9a-1 from the venom of the sea anemone *Urticina grebelnyi* [81] and PhcrTx1 from *Phymanthus crucifer* [82] have been reported to target ASIC channels. These peptides are cross-linked by two disulfide bridges and have no sequence homology to other sea anemone neurotoxin peptides.

#### 5.3.5. TRPV1 Inhibitors

TRPV1s are non-selective cation channels expressed in mammalians peripheral and central neuronal systems. They initiate neuronal response during inflammation stimuli, which allow them to be regarded as one of the most important molecular triggers of pain stimuli. The first peptidic TRPV1 inhibitor isolated from sea anemone venom was τ-SHTX-Hcr2b (APHC1) from *Heteractis crispa* [83]. Subsequently, two homologous peptides (τ-SHTX-Hcr2c (APHC2) and τ-SHTX-Hcr2d (APHC3) that target TRPV1 were isolated from the same species [84]. These novel neurotoxins are promising new models for designing a new generation of analgesic drugs.

### 5.4. Non-Protein Bioactive Components

In addition to the protein and peptide compounds mentioned above, a number of pharmacologically active low molecular weight compounds have been detected in cnidarian venoms. Large amounts of 5-hydroxytryptamine (5-HT, serotonin) have been reported in the water where hydra was stimulated electrically [85]. This presumably nematocyst-derived substance causes instant pain in predators and thus may have a defensive role [85]. Also the vasodilatation enhanced by serotonin may potentiate the effect of other venom components [85]. Histamine has been detected in the homogenised tentacles of the sea anemones *Anemonia viridis* (previously known as *Anemonia sulcata*) and *Actinia equina* [86,87]. Like serotonin, histamine produces sharp pain and increases vascular permeability. Although these compounds have not been directly detected in isolated nematocysts, it is likely that they are involved in cnidarian envenomation, and treatment of sting victims with antihistamines has been recommended to relieve the symptoms [125]. Bunodosine, an *N*-acylamino acid, was purified from the venom of the sea anemone *Bunodosoma cangicum*. This compound exhibits a potent analgesic activity by activation of serotonin receptors [88]. Caissarone is a quaternary purine derivative isolated from the sea anemone *Bunodosoma caissarum* by extracting the whole animal in acetone. This marine product has a high antagonist activity towards guinea-pig ileum adenosine receptors which stimulate gut function [89].

## 6. The Role of Cnidarians Venoms in Drug Discovery

Toxic compounds isolated from Cnidaria have been viewed to produce several serious implications to human health due to their neurotoxicity, cytotoxicity and tissue damage. However, novel findings have demonstrated that their toxins might offer a tool to study cell physiology [126] and provide promising sources of pharmacological lead/active agents for therapy of human diseases.

Palytoxin is a highly potent non-protein toxin isolated from order Zoantharia (soft corals) of the genus *Palythoa* and *Zoanthus* and the sea anemone *Radianthus macrodactylus* [127]. The interaction of palytoxin with Na^+^, K^+^-ATPase pump in almost every excitable tissue induces passive ion conductance which triggers K efflux, Na influx and massive membrane depolarization and tissue contraction [127,128]. Palytoxin has been reported for anti-cancer activity against head and neck carcinoma cells [129], Ehrlich ascites tumour and P-388 lymphocytic leukaemia cells [130]. The mechanism of action of its cytotoxicity and tumour suppressor activity has been established by actin filament distortion and apoptosis [131]. Conversely, palytoxin has been identified as a tumor promoter by disrupting the regulation of cellular signalling cascades [132].

Over the past decade, several cytolysins and protease inhibitors have been extracted from the sea anemone *A. equina*. Equinatoxin II (EqT II) is a pore-forming protein that has been shown to have significant toxicity against Ehrlich ascites tumour and L1210 leukaemia cell lines [133] and diploid lung fibroblasts of the Chinese hamster [134]. Equistatin is a potent inhibitor of papain-like cysteine proteinase and aspartic proteinase cathepsin D [135]. Overexpression and hypersecretion of cathepsin-D has been reported in breast carcinoma cells [136], and papain-like cysteine proteases are involved in diseases of the central nervous system [137].

Recently antibutyrylcholinestrasic activity was detected in the crude venom extracted from the tentacle material of the Mediterranean jellyfish *Pelagia noctiluca*. Inhibition of butylcholinestrase in the central nervous system may prove useful in the treatment of neurodegenerative diseases such as Alzheimer’s disease and senile dementia [138].

ShK is a potent Kv1.3 channel blocker toxin that isolated from the sea anemone *Stoichactis helianthus*. Since this channel is crucial in the activation (proliferation and cytokine production) of human effector memory Tcells (T_EM_), ShK could provide a valuable immunosuppressant for the treatment of autoimmune diseases mediated by T cells [73]. Kv1.3 blockers are also considered as a therapeutic target for the treatment of obesity, thus highlighting the potential use of ShK in treatment of obesity and insulin resistance [74].

## 7. Envenomation

Fortunately, due to the biophysical properties of the discharge event many cnidarians lack the capacity to penetrate human skin. Further, stings by most species with cnidae capable of perforating human tissue lead only to a negligible to moderate transient irritation/burning sensation. In contrast, contact with several jellyfish, sea anemone and coral species can cause severe pain, tissue damage and even cardiovascular collapse and death. The venom of the hazardous animals is introduced into the target tissue (epidermis, dermis, vasculature, lymphatic system and probably in some cases subcutaneous or muscular structures) (Figure 1) [125] and initiates immediate and delayed immunological and toxicological responses [139,140,141]. Envenomation symptoms are determined by the contents of the venom, the volume of the injection, the health of the patient and the duration of the tentacle-skin contact [125].

One of the most important steps in the treatment of human envenomation is to use specific fluids to prevent further nematocyst discharge, since physical attempts to detach remaining tentacles from the victim’s skin might cause massive discharge [34,142]. Since 1908 numerous traditional remedies such as urea, seawater, vinegar, methylated spirits etc. have been used to inactivate the undischarged nematocysts on the adhered tentacles and/or to alleviate pain. These chemicals have been used to varying degrees of success, ranging from complete success in certain species to complete failure in others. For instance, while certain chemicals such as ethanol, or 5% acetic acid in distilled water, cause massive nematocyst discharge in Hydrozoa and Cubozoa species [14,143,144], some other such as a food grade vinegar was found to inactivate the penetrating nematocysts of *C. fleckeri* rapidly and completely [34].

Avoidance and treatment of human envenomation by cnidarian species is an increasingly important objective within the broader scientific community. It also represents a collaborative aim of medical, pharmacological, toxicological and biological disciplines towards developing improved understanding of field ecologies as well as effective therapeutics to minimize pathogenic impact on bathers. It also can improve our understanding of the food web and biome dynamics of these ancient and intriguing animals.

## 8. Conclusions

Members of Cnidaria phylum are ancestral venomous Eumetazoan with a unique phylogeny. In spite of recent efforts to extract and characterize novel toxins from cnidarians, much more remains to be done to investigate their toxins and their potential as new sources of therapeutic substances. Of the approximately 10,000 cnidarian species, only 156 toxins from 10 pharmacological families have been defined. Potentially useful new toxicological information is gradually accumulating. One promising solution towards broadening the knowledge on cnidarian toxins is to apply high throughput sequencing technologies to identify novel structural and pharmacological groups of toxins [145,146]. Nevertheless, the enormous potential of cnidarian venoms for understanding the activity of different receptors and channels in health and disease conditions, as well as their use as sources of novel pharmaceutical lead compounds, appears to offer endless possibilities for future scientific research.

## Abbreviation

rDNA: ribosomal DNA
mtDNA: mitochondrial DNA
PLA_2_: Phospholipase A_2_
MACP: Membrane Attack Complex-Perforin
SCRiPs: Small cysteine-rich peptides
